# Abnormal sensation during total body irradiation: a prospective observational study

**DOI:** 10.1093/jrr/rrac042

**Published:** 2022-07-11

**Authors:** Masashi Mizumoto, Yoshiko Oshiro, Toshio Miyamoto, Taisuke Sumiya, Shosei Shimizu, Takashi Iizumi, Takashi Saito, Hirokazu Makishima, Haruko Numajiri, Kei Nakai, Toshiyuki Okumura, Takeji Sakae, Kazushi Maruo, Hideyuki Sakurai

**Affiliations:** Department of Radiation Oncology, University of Tsukuba, Tsukuba, Ibaraki 305-8576, Japan; Department of Radiation Oncology, University of Tsukuba, Tsukuba, Ibaraki 305-8576, Japan; Department of Radiation Oncology, Tsukuba Medical Center Hospital, Tsukuba, Ibaraki 305-8576, Japan; Department of Radiation Oncology, University of Tsukuba, Tsukuba, Ibaraki 305-8576, Japan; Department of Radiation Oncology, University of Tsukuba, Tsukuba, Ibaraki 305-8576, Japan; Department of Radiation Oncology, University of Tsukuba, Tsukuba, Ibaraki 305-8576, Japan; Department of Radiation Oncology, University of Tsukuba, Tsukuba, Ibaraki 305-8576, Japan; Department of Radiation Oncology, University of Tsukuba, Tsukuba, Ibaraki 305-8576, Japan; Department of Radiation Oncology, University of Tsukuba, Tsukuba, Ibaraki 305-8576, Japan; Department of Radiation Oncology, University of Tsukuba, Tsukuba, Ibaraki 305-8576, Japan; Department of Radiation Oncology, University of Tsukuba, Tsukuba, Ibaraki 305-8576, Japan; Department of Radiation Oncology, University of Tsukuba, Tsukuba, Ibaraki 305-8576, Japan; Department of Radiation Oncology, University of Tsukuba, Tsukuba, Ibaraki 305-8576, Japan; Department of Biostatistics, Faculty of Medicine, University of Tsukuba, Tsukuba, Ibaraki 305-8575, Japan; Department of Radiation Oncology, University of Tsukuba, Tsukuba, Ibaraki 305-8576, Japan

**Keywords:** radiotherapy, light flash, odor, total body irradiation (TBI), observation study, prospective

## Abstract

Light flash and odor during radiotherapy are well-known phenomena. Two prospective observational studies have indicated that 55% of patients observed a light flash during irradiation of the retina and 27% of patients sensed an odor during radiotherapy for the nasal cavity. A prospective observational study was performed in all patients at our hospital who received total body irradiation (TBI) between January 2019 to October 2021. Light flash and odor during TBI were examined using the same method as that used in previous studies. A total of 32 patients received TBI during the study period. The patients had a median age of 41 (18–60) years, and included 20 males and 12 females. A survey checklist showed that 14 patients (44%) sensed light and 14 patients (44%) sensed odor during TBI,. The color of the light during irradiation was yellow in six cases, white in four cases, and blue in four cases. The intensity of the light was 2–5 (median 3, 1 is very weak, 5 is very strong) and the time over which the light flash was felt was 4–60 s (median 10 s). Two patients each sensed smells of plastic, ozone and bleach, and others sensed one smell each. The intensity of the odor was 1–4 (median 3, 1 is very weak, 5 is very strong) and the time over which the odor was sensed was 1–25 s (median 3 s). We conclude that light flashes and odors are each sensed by 44% of patients during TBI. Various types of light flashes and odors were reported in this study.

## INTRODUCTION

Light flash and odor during radiotherapy are well-known phenomena among radiation oncologists. Two prospective observational studies have indicated that 56% of patients sensed a light flash during retina irradiation and 27% of patients sensed an odor during nasal cavity irradiation [1, 2]. In our previous research, light flash and odor were felt with both X-ray and proton beam therapy, and pediatric patients (under 20 years old, median 10 years old) also sensed a light flash and odor with a certain probability (data not shown).

Total body irradiation (TBI) is performed as a pretreatment for umbilical cord blood transplantation, hematopoietic stem cell transplantation, and peripheral blood stem cell transplantation. In TBI, the whole body is exposed to X-rays, including the entire eyeball and nasal cavity. The dose-rate of TBI used in this study was 75 MU/min, which is lower than those in photon therapy (400–600 MU/min) and proton therapy (1300 MU/min). Here, we focus on light flash and odor during TBI.

## PATIENTS AND METHODS

The study was performed in our hospital from January 2019 to October 2021, and was approved by the institutional review board (R01-160, Tsukuba Clinical Research & Development Organization). TBI was performed at our hospital. All patients who received TBI in the study period were included, except for those who could not communicate and those with visual abnormalities or abnormal olfactory sensations. Visual abnormality was defined as unable to distinguish the colors on the chart with or without glasses, and abnormal olfactory sensation was confirmed by interview. A checklist was used with the same format as in previous studies to evaluate light and odor during TBI [1, 2]. TBI was performed at 12 Gy in 6 fractions for 3 days, and TBI on the same day was performed with an interval of at least 6 hours. TBI at our hospital is administered at 20 min per irradiation, including 10 min each in the supine and prone positions. TBI is performed using a mobile bed that turns around twice over one irradiation. One-way irradiation for about 15 to 20 s is performed on the eyeball and nasal cavity, with a total of about 1 min of irradiation applied to the eyeball and nasal cavity. In principle, a lens block is not used, but a lung block for 4 Gy is used and the lung dose is reduced to 8 Gy.

### Statistical analysis

The presence or absence of a smell or light flash was evaluated using multiple logistic regression with gender and age as explanatory variables. *P* < 0.05 was considered to be significant. All statistical analyses were conducted with SAS ver. 9.4 (SAS Institute Inc., Cary, NC).

## RESULTS

A total of 32 patients who received TBI were examined. The characteristics of these patients are shown in Table 1. Fifteen patients had chronic myeloid leukemia, 13 had acute lymphocytic leukemia and four had other diseases (myelodysplastic syndrome, NK/T-cell lymphoma, T-cell lymphoma and diffuse large B-cell lymphoma). TBI was performed as a pre-transplantation treatment (21 cord blood transplants, seven hematopoietic stem cell transplants, and four autologous peripheral blood stem cell transplants). The checklist was used only once during the TBI period on the 2nd to 6th days, including on the 6th day in 19 of the 32 cases.

**Table 1:** Patient characteristics

Characteristics	*n* = 32
Age (years)	18–60 (range) 41 (median)
Gender	
Male	20
Female	12
Feel Light Flash	
Yes	14
No	18
Feel odor	
Yes	14
No	18

The patients had a median age of 41 (18–60) years, and included 20 males and 12 females. The survey checklist showed that 14 patients (44%) sensed light and 14 patients (44%) sensed odor during TBI. The details of the light flashes and odors are shown in Table 2. The color of the light during irradiation was yellow in six cases, white in four cases, and blue in four cases. The intensity of the light was 2–5 (median 3, 1 is very weak, 5 is very strong) and the time over which the light was felt was 4–60 s (median 10 s). Four of 14 patients sensed light only when in the prone position.

**Table 2:** Details of abnormal sensations in each patient

Patient	Age	Gender	Light Flash (Color / Intensity / Time)	Odor (Type / Intensity / Time)	Comment
1	19	F	None	Rubber / 4 / 3	None
2	60	M	White / 2 / 15	None	None
3	46	M	Yellow / 4 / 60	None	None
4	50	M	None	Bleach / 3 / 3	None
5	26	M	None	Alcohol / 4 / 3	None
6	52	F	Yellow / 4 / 25	None	Felt only prone position
7	28	M	None	Refreshing / 2 / 2	None
8	18	M	Yellow / 3 / 4	Chlorine / 2 / 4	None
9	48	M	White / 4 / 10	Plastic / 1 / 1	None
10	47	F	Blue / 4 / 5	None	Felt only prone position
11	45	F	Yellow / 3 / 5	None	None
12	40	F	None	None	None
13	42	M	None	None	None
14	24	M	None	Bleach / 4 / 5	None
15	27	M	None	None	None
16	38	M	None	Metal / 2 / 2	None
17	52	F	None	None	None
18	37	M	Blue / 3 / 10	None	None
19	50	F	None	Disinfectant / 3 / 5	None
20	42	M	White / 3 / 30	None	
21	44	M	Blue / 4 / 15	Ozone / 4 / 25	Felt light flash only prone position
22	26	M	None	None	None
23	23	F	None	None	None
24	52	M	None	None	None
25	39	F	None	Smoke / 3 / 10	None
26	52	F	None	None	None
27	50	M	None	None	None
28	41	M	White / 5 / 10	None	None
29	38	F	Yellow / 3 / 10	Burnt / 2 / 2	Felt light flash only prone position
30	25	M	Blue / 3 / 4	Ozone / 2 / 2	None
31	48	M	None	None	None
32	27	F	Yellow / 2 / 5	Plastic / 3 / 3	None

There were also various types of odors during irradiation, with two patients each sensing the smell of plastic, ozone, and bleach, and all other smells were sensed in one case (Table 2). The intensity of the odor was 1–4 (median 3, 1 is very weak, 5 is very strong) and the time over which the odor was sensed was 1–25 s (median 3 s). The duration over which patients recognized light and odor was dependent on their sensory response and an accurate time was not measured. In multiple logistic regression analysis, age (OR = 0.920, *P* = 0.026) was significantly associated with a sense of smell (Table 3).

**Table 3:** Multiple logistic regression analysis of presence of a sense of light or smell

Variable	Odds ratio	95% CI	*P* value
Sense of light			
Gender (female/male)	0.840	0.186–3.612	0.815
Age	1.021	0.960–1.092	0.511
Sense of smell			
Gender (female/male)	1.011	0.197–5.196	0.990
Age	0.920	0.848–0.985	0.026

## DISCUSSION

All 32 cases in this study were treated with the same irradiation method, and the data collected reflect light flash and odor during irradiation when the entire eyeball and entire nasal cavity are irradiated. Previous studies [3–9] have commonly shown that light flash and odor may be sensed during radiotherapy to the brain, head and neck. To examine light flash in more detail, we conducted a prospective observational study of adults who received radiation or proton therapy, and found that light flash during irradiation was sensed in 56% and 6% of cases in which the retina was and was not in the irradiation range, respectively [1].

In the current study of TBI, the entire retina was irradiated and light flash was sensed in 14 of 32 cases (44%). The reason for the lower rate of sensing a light flash despite the entire retina being irradiated is unclear, but the result may statistically be within the margin of error and the dose-rate per unit time is lower in TBI. In our previous study, the color of light flash was blue or purple in most cases in which the retina was irradiated, and the proportion of yellow was high in irradiation of the trunk for patients in whom the retina was not irradiated [1]. The color of the light during TBI was mainly yellow, which tended to be similar to that in irradiation of the trunk. The difference between TBI and conventional radiotherapy or proton beam therapy is that TBI irradiates the entire retina, but the amount of irradiation per time is small. From the above results, it seems that yellow light is often felt in irradiation of the trunk or in TBI with less irradiation of the retina per time, and blue or purple light is more common with a higher amount of irradiation per time. Irradiation was performed in two positions, supine and prone, but four of 14 cases sensed light only when prone. The reason for sensing light flash only in the prone position is unknown, but the surroundings may appear darker and the irradiation dose may increase due to scatter from the bed when in a prone position.

In previous studies, 27% of patients in whom the nasal cavity was included in the radiation field sensed odors and only 5% of patients in whom the nasal cavity was not included in the radiation field sensed odors [2]. An odor was sensed in 44% of TBI cases in the current study, which is a higher rate than that in radiotherapy other than TBI. It is hypothesized that odor occurs due to ozone generated during irradiation [7, 10, 11]. Although the irradiation per unit time for TBI is small, the amount of ozone generated in the nasal cavity may be large because the entire nasal cavity is included in the irradiation range. In photon radiotherapy or proton beam therapy, 63% of the odors were burnt odors and 16% were chemical odors, but TBI produced various odors, including burnt odors and chemical odors. In two of the 14 patients who sensed an odor during TBI, the type of odor was described as that of ozone in the free description, which may support the odor during radiation therapy being that of ozone generated by irradiation. However, there are various types of odor descriptions and it is difficult to identify the causative substance only by the type of odor. Recently, Kosugi *et al.* reported that a patient whose olfactory epithelium was completely resected sensed a smell during radiotherapy and concluded that the central nervous system (CNS) may have detected X-rays directly [12].

Since TBI includes irradiation twice for 15 to 20 s each in the supine and prone positions, the irradiation time of the eyeball and nasal cavity is at least 1 minute in total in one irradiation. However, the total median time over which light and odor were actually sensed in this study was 10 s or less, and thus, light flash and odor were not sensed over the whole irradiation time. In the survey, some patients stated that they recognized light and odor coincidentally at the start of irradiation, and it is possible that light and odor were detected for a moment at the time of starting irradiation. However, the time over which patients sensed light and odor was not clearly consistent with the radiotherapy duration. Since ozone is unstable and the amount of ozone generated is small, we speculate that ozone may be smelled only at the first moment when the nasal cavity is irradiated. Light may not be perceptible due to the brightness of the room and may not be perceived except at the time of starting irradiation. In some cases, light flash is felt for a relatively long time compared to odor, and it may be easier to sense light compared to odor. We have also found in previous studies that younger patients are more sensitive to light and odor [1, 2], and a similar result was found in the current study. The reasons for this observation are unclear, and significant differences were unlikely to be shown because of the small number of younger patients receiving radiotherapy.

In conclusion, about 40% of patients felt light flash and odor during TBI. The properties of light flash differ between normal radiotherapy and TBI, and it is possible that the amount of irradiation per unit time affects these properties. We are currently planning a more detailed prospective study to investigate light flash and odor during irradiation.

## CONFLICT OF INTEREST

None.

## FUNDING

This work was supported by the University of Tsukuba.
